# Cognitive Loading and Knowledge Hiding in Art Design Education: Cognitive Engagement as Mediator and Supervisor Support as Moderator

**DOI:** 10.3389/fpsyg.2022.837374

**Published:** 2022-02-25

**Authors:** Tao Gao, Lihong Kuang

**Affiliations:** ^1^Academy of Fine Arts, Jiangxi Normal University of Science and Technology, Nanchang, China; ^2^Faculty of Decorative Arts, Silpakorn University, Bangkok, Thailand; ^3^Department of Art Design, Gongqing College of Nanchang University, Gongqingcheng, China

**Keywords:** cognitive loading, cognitive engagement, knowledge hiding, supervisor support, Art design trainees

## Abstract

The aim of the study is to document a new predictor of knowledge hiding from the perspective of Art design trainers and Art design trainees in virtual training’s and this study tends to add new theoretical insights into the body of literature. For this purpose, this study approached a sample of 500 respondents under a cross-sectional research design and respondents who have participated in virtual trainings or their trainings were at the final stage were recruited through the snowball sampling technique. The useable responses remained at 406 and these have been analyzed through SPSS for demographic analysis and Smart-PLS has been used to test the structural model, while a process macro has been used to test the moderation. Results indicate that cognitive loading has the potency to reduce the knowledge hiding behavior of the trainees. Similarly, it has been observed that cognitive loading increases the cognitive engagement of the trainees, and it moreover reduces the knowledge hiding tendency of trainees. In case of mediation, a partial mediation has been documented through the variance accounted for (VAF) approach while testing moderation. The role of supervisor support has not been found to be statistically significant.

## Introduction

In the search of “impacts of cognitive loading on knowledge hiding in virtual educational trainings,” it has been important for going deep into the cognitive loading and its settings. The idea is derived from cognitive load theory and management of cognitive load. Knowledge sharing and knowledge hiding are two opposites sides which are determined by various psychological and management factors (Wu et al., 2022). It is proven that knowledge hording or hiding is influenced by many factors especially in Art design teaching whether it be physical or online. Three different types of knowledge hiding are reported by Connelly et al. (2012) including evasive one, playing dumb, and rationalized knowledge hiding. Rationalized hiding is referred to as justified or justification-based knowledge hiding. Providing justifications to the planned activities is a cognitive aspect of psychology (Rong and Liu, 2021). A relational aspect could be developed between cognitive loading and such kind of knowledge hiding. Cognitive loading is actually a management of working memory as defined by the cognitive load theory due to which certain things occupy the segment of brain that acts in delivering the knowledge to learners. Due to the onset of the pandemic, a worldwide shift to online teaching and learning has been witnessed. So, the impact of all cognitive factors became necessary to be analyzed in such Art design virtual educational contexts virtual educational contexts.

Dissecting the cognitive load, first it is important to understand the cognitive load theory given by Sweller (1994). According to the theory, cognitive load is the capacity of the brain related to working ability defined by him. The part of our brain or the working memory that analyzes what we are doing right now can only handle a certain amount of data at a time. According to the theory, there are three types of cognitive load which should be understood. Among them, the basic one is intrinsic load, then the extrinsic load, and the last one is the germane load (Sweller, 1988). Intrinsic load refers to a task that can be difficult at times. Compare the difference between calculus and arithmetic. Maybe one who is good at math would not find it too difficult. For others, this endeavor would need a high level of mental focus. While extrinsic load is somewhat different in which the tutor has more control over learners. It is caused by the communication of ideas that have nothing to do with the assignment. Germane load refers to information processing in which organization of information in the brain takes place. It is affected by the information previously learned (Sweller, 1988).

According to cognitive load theory, the amount of pressure placed on learning and memory by the educational topic has a significant impact on whether a learner succeeds in learning (Bai, 2021). According to a basic concept, working memory has a limited capacity, while long-term memory has a considerably larger capacity since information is stored in schemas. As a result, working memory becomes a bottleneck, requiring educators to provide learning content that maximizes the quantity of knowledge kept in long-term memory (Nagy Kem, 2016). The cognitive load theory explains that it was a one-dimensional term which only applied to overall cognitive functioning, and there is still debate over how to conceive different forms of cognitive load (Ayres, 2018). The ability to hold important, unusual, and complicated knowledge that has been identified as a cornerstone to provide educational organizations with a long-term competitive advantage (Stiller and Bachmaier, 2018; Skulmowski and Xu, 2021). Because knowledge is dispersed asymmetrically inside organizations, facilitating efficient knowledge sharing methods across stakeholders is critical to establishing competitive advantages. This means that the effectiveness of knowledge management is dependent to a considerable extent on people’s willingness to share their knowledge. As a result, academics have looked at the elements that impact people’s willingness to share their expertise in diverse social circumstances (Zutshi et al., 2021).

This referencing leads to the development of a connection between cognitive loading and knowledge hiding meaning that there could be an effective relationship between cognitive load and knowledge hiding. This is due to the psychological nature of both variables as in cognitive load management by the teacher and learners force them to hide or share the knowledge in any context of physical or online learning. This relationship is suggested by a few researchers (Hsiao et al., 2013) which allowed us to investigate such a relational study. This kind of relationship could be mediated by a cognitive factor such as cognitive engagement. Emotional, behavioral, and cognitive involvement are three fundamental components of engagement, which make it a multifaceted entity (Hsiao et al., 2013; Xie et al., 2019). The literature on school involvement, with an emphasis on educational failure and change, informs engagement research. We have seen a growing emphasis on student participation at the individual level, in specialized learning or problem-solving situations, in recent years. Being thoughtful, experiencing flow, and perseverance all reflect cognitive engagement, which is defined as the amount of mental involvement in learning (Lyakhova et al., 2021). There is some evidence that there is a link between cognitive involvement and student achievement (Wolters and Taylor, 2012; Boekaerts, 2016).

The mediating role of cognitive engagement has been examined in previous research such as Jelas et al. (2016) which suggested its role in the context of our study that it could mediate the relationship between cognitive loading and knowledge hiding. The degree to which students are eager and allowed to carry on the learning job at hand is referred to as cognitive engagement (Corno and Mandinach, 1983; Appleton et al., 2006). This includes how much effort pupils are willing to put into the activity and how long they are willing to work on it. The level of students’ homework, class participation, supplementary involvement in events, or general contact with teachers, as well as how motivated they appear when participating in classroom discussions, has traditionally been used to measure cognitive engagement. Most writers believe cognitive involvement to be a more or less stable quality of students, regardless of the setting, based on this description.

Working in groups and participating in conversations, looking for information on the Internet, or listening to a lecture are likely to result in varying levels of cognitive involvement due to varying amounts of autonomy. Listening to a lecture is considered the least intellectually engaging because there is little to no student agency in such situations. When students autonomously look for information on the Internet, or participate in self-initiated information-seeking behaviors, the level of autonomy should be relatively high, leading to increased cognitive engagement. Depending on the dynamics of the group, working in groups and participating in debates might result in high or low emotions of autonomy. In contrast to a group that works well together, a student may feel less independent and participate less cognitively if there are dominant classmates in the group (Rotgans and Schmidt, 2011). Based on this evidence, we utilized cognitive engagement as a mediator in our study model.

In the whole scenario of cognitive loading, cognitive engagement, and knowledge hiding, there was a need to identify some moderating roles. In this regard, supervisor support provided us with a hint of a regulating role in such relationships. Such kind of regulatory statues are suggested by many researchers of the past such as Bai (2021), Rong and Liu (2021), Wen and Ma (2021), and Wu (2021). The function of a supervisor has grown more vital than ever before in today’s highly competitive and dynamic corporate climate. Supervisors are crucial to an organization’s success because they engage, motivate, and retain personnel. In today’s enterprises, supervisors play a critical role. According to the findings, supervisors not only represent the organization, but also partially replace it in terms of keeping their staff engaged and eager to stay (Jelas et al., 2016). Supervisor support has also been linked to a number of positive organizational outcomes, including increased educations organizational commitment, performance, satisfaction, and role clarity (Agarwala et al., 2014). Such kind of support provided us the opportunity to investigate the moderating role of supervisor support in regulating the role of cognitive loading toward knowledge hiding mediated by cognitive engagement. This study revolved around given circumstances with the following objectives as: (1) To assess the role of cognitive loading and cognitive engagement. (2) To analyze the mediating role of cognitive engagement between cognitive loading and knowledge hiding. (3) To explore the moderating impact of supervisor support among cognitive loading, engagement, and knowledge hiding.

## Review of Literature and Hypothesis Development

This study model is based on two cognitive theories which suggest influential roles of cognitive load and cognitive engagement in the context of knowledge hiding behaviors. First, the variable cognitive loading is derived from cognitive load theory and the cognitive engagement from integrated cognitive antisocial potential (ICAP) theory of cognitive engagement for the load management and knowledge hiding management.

### Cognitive Load Theory

Sweller (1988) established the cognitive load theory in the disciplines of education and instructional design, based on a model of working memory. According to cognitive load theory, information storage and processing are based on two interdependent systems. Human working memory is responsible for knowledge processing, whereas the long-term memory is responsible for storing data in the form of schemata. Cognitive load theory is a branch of schema theory that explains how people learn and store knowledge by combining lower-order and higher-order schemata (Wu et al., 2022). Working memory resources are finite, according to cognitive load theory, and processing and retaining information consumes a portion of these resources. As a result, once a certain quantity of data is stored in a single schema, it may be kept in working memory at a lower cognitive cost. The cognitive load is viewed as a multi-factorial term in cognitive load theory, with various sources of cognitive burden creating varied loads.

Sweller differentiates three types of cognitive stress. Intrinsic load is determined by the amount and interactivity of components that must be processed and is directly tied to the learning content. Anything that must be or has been learned, including a notion or procedure, is specified as an element. The term “interactivity” refers to how elements interact with one another (Sweller, 2010). A collection of chemical symbols, for example, might be considered low interactive material since these elements are not dependent, but learning to calculate second degree problems is considered high interactive. Intrinsic load is determined by the student’s degree of skill since the more knowledgeable they are, the more information may be shrunk onto high-order schemata, lowering the cognitive cost of keeping pieces in working memory. Extraneous load focuses on the mental resources used for things that do not help with learning, schemata acquisition, or automation.

The mental resources committed to collecting and automating schemata in long-term memory are referred to as germane load. Sweller came up with the notion of cognitive load after seeing that some educational forms might also boost cognitive burden and learning (Ayres, 2006). If superfluous load must be decreased to prevent exhausting working memory resources, relevant load must be emphasized to improve learning. Cognitive load theory may explain how an increase in cognitive load might benefit the work at hand rather than being primarily tied to a decrease in performance. The triarchic model of cognitive load is now the subject of heated dispute in the cognitive load theory field. Researchers disagree on how the loads should be conceptualized as well as the nature of the interactions between them (Schnotz and Kürschner, 2007; de Jong, 2010).

According to the researchers, intrinsic load relates to task performance, whereas germane load refers to additional cognitive processes that might improve learning, such as the intentional use of a learning technique. In other words, they argue that while learning may occur without relevant load, it can be enhanced by it. Some studies go even farther, claiming that germane and intrinsic loads are two ideas that are interchangeable. Because there is no need to refer to relevant load to explain the primary consequences predicted by cognitive load theory, he views it as a theoretical construct without empirical support (Kalyuga, 2011). Since the role of cognitive load in sharing the knowledge or hiding has been established through cognitive load theory and its types, cognitive loading was used as a variable in light of this theory.

### ICAP Theory of Cognitive Engagement

According to the research, cognitive engagement, as it is presently measured and characterized, appears to promote only shallower learning. The ICAP theory was created to address these two issues by defining cognitive engagement or student engagement (the terms are related in ways that might improve deeper learning). The ICAP theory was initially published in 2009 (Chi, 2009), in a study that hypothesized three cognitive modes of interaction (Active, Constructive, and Interactive), as well as data from the literature to back up ICAP’s predictions that Interactive is floating between Constructive and Active. Because multiple laboratory and classroom investigations described in the report compared the passive mode to one of these three alternative active modes, ICAP was expanded in 2014 to incorporate the passive mode (Chi and Wylie, 2014). As a result, whereas the term active in ICAP refers to one form of interaction, the term active in active learning refers to all three kinds of cognitive involvement (Menekse et al., 2013).

ICAP is made up of three parts: a taxonomy of four engagement modes with operational definitions for each, a metric for defining the degree of engagement based on the cognitive processes associated with the four behavioral modes, and a hypothesis for predicting the hierarchical levels of student learning as a function of the mode of engagement. Findings from published research in the literature are used to back up the theory’s predictions (Chi, 2009; Chi and Wylie, 2014; Chi et al., 2018). The theory provides the basis for cognitive engagement and its measurement in numerous cases of learning. In the context of cognitive loading effects on knowledge hiding behaviors, this theory could be applied to find the role of cognitive engagement of the students in the learning process. The mediating link of cognitive engagement could be easily determined by the support of this theory. So, we utilized the information provided by Chi (2009) in identifying the role of cognitive engagement in this study.

### Relationship Between Cognitive Loading and Knowledge Hiding

We hypothesized that the role of cognitive loading leads to a significant relationship with knowledge hiding. The load that completing a task places on the learner’s cognitive system is known as cognitive load (Paas and Van Merriënboer, 1994). According to this theory, the objective, the requisite mental representations, the learner’s inventory of cognitive schemata, and processing techniques influence learning processes that lead to knowledge creation and automation. The cognitive burden placed on the learner’s working memory by performing learning activities and accompanying learning processes is significant. The contrast between intrinsic load, which is caused by the work, and external burden, which is caused by poor teaching, is at the heart of the cognitive load hypothesis. Intrinsic load is a type of activity dictated by the nature of the task demands in relation to the learner’s skill and motivation. Extraneous load, which is ineffectual for learning, and germane load, which is helpful for learning, are both possible outcomes of instructional design. Extraneous cognitive burden is defined as extra cognitive strain that is unnecessarily added because of poorly designed education. Load that adds to learning, such as self-explanations, is referred to as germane load (Paas and Van Merriënboer, 1994).

According to the literature on knowledge management, knowledge grows as it is used (Rong and Liu, 2021; Wen and Ma, 2021). According to the researchers, information is important when it is shared with others (Giustiniano et al., 2016; Škerlavaj et al., 2018). While information sharing among individuals benefits a company, many people are uncomfortable or hesitant to share their expertise with their coworkers, which can stifle creativity. Despite the fact that people are aware that sharing information benefits the larger society, they nonetheless weigh the financial cost of contributing, such as the fear of losing power or position, or the fear of being underestimated (Fong et al., 2018). As a result, many people do not genuinely share all their information. Individuals may, on the other hand, participate in knowledge concealment. In other words, when their colleagues ask them to share information, they try to keep it hidden or withhold it. In academia and research institutes, the human proclivity to scrutinize their own expertise with caution is a serious element (Babic et al., 2019).

To summarize, knowledge sharing or knowledge hiding in virtual educational trainings imposes additional unnecessary strain without assistance that deals with varied group composition and online communication. Because extra cognitive resources must be committed to identifying a good partner and learning how to engage with others online, this is the case. These organizing and sustaining processes reduce the cognitive capacity available for knowledge formation because, according to cognitive load theory, different types of cognitive load build up and there is an upper limit to the available cognitive load defined by working memory restrictions (Babic et al., 2019). So, based upon these analogies, a relationship was suggested between cognitive loading and the behavior of knowledge hiding, and we suggested the following hypothesis for analysis for new levels of virtual educational training context.

***H_1_.*** *There is a significant relationship between cognitive loading and knowledge hiding.*

### Relationship Between Cognitive Loading and Cognitive Engagement

The question of student engagement in higher education has been a hot topic in educational research circles. This is due to its link to desirable learning outcomes such as critical thinking and grades, educational quality, and student success measures such as academic achievement (Robinson, 2008; Becker et al., 2009). Research on cognitive involvement in the setting of virtual education has lately been published. According to an empirical study, student engagement in a virtual reality learning environment was much higher than in a typical asynchronous learning platform (Carini et al., 2006; Claman, 2014). Cognitive load, according to Paas and Van Merriënboer (1994), is a multi-dimensional concept that indicates the stress that doing a certain activity places on a learner’s cognitive system. According to cognitive load theory, a high mental burden necessitates the deployment of more resources for data entry. Other researchers have looked at the effects of physical and perceived cognitive strain.

An electroencephalogram was used in a clinical trial to track degrees of task engagement and mental effort in vigilance, learning, and memory activities. Electroencephalogram measurements were discovered to have a link to both subjective and objective performance markers. They also discovered that when the task demands rose, task engagement and mental workload electroencephalogram-based indicators increased correspondingly (Berka et al., 2007). Other researchers (Berka et al., 2007) also validated these findings, claiming that higher levels of immersion produced by virtual worlds resulted in higher electroencephalogram evaluations of cognitive load. Virtual reality experiences have been proven to have a favorable influence on the cognitive load associated with the same activity in real life (Andersen et al., 2015; Lackey et al., 2016). Authors have, on the other hand, documented considerable increases in cognitive load because of interacting with virtual reality settings. For example, researchers found that when virtual presence grew, cognitive load increased, but students’ retention and understanding improved as well (Schrader and Bastiaens, 2012). The relational aspect of cognitive loading and cognitive engagement allowed us to analyze the relationship between both through the following hypothesis.

***H_2_.*** *There is a positive relationship between cognitive loading and cognitive engagement.*

### Mediating Role of Cognitive Engagement Between Cognitive Load and Knowledge Hiding

A significant discussion about cognitive engagement and cognitive loading has been done in the preceding part of the manuscript which suggested a strong relationship between both. It is worth mentioning that there could be a negative relationship between cognitive engagement and knowledge hiding and more engagement would yield into more knowledge sharing among each other whereas it would lead to lesser knowledge hiding. Therefore, it was necessary to check the mediating association of cognitive engagement between both factors of cognitive loading and knowledge hiding. A number of studies in the past have evaluated the mediating role of cognitive engagement in different perspectives such as reported by Cheng (2013) and Raja Kamal et al. (2021). But no research investigated the mediating link of cognitive engagement between cognitive loading and the knowledge hiding which yielded a research gap for us.

The research conducted by Raja Kamal et al. (2021) suggested that cognitive engagement positively played a mediating role between the online teaching and the understanding and learning in the said setup. The research conducted by Joo et al. (2017) suggested that cognitive engagement showed partial mediation. It was indicated that employees’ perceived work cognitions accounted for 31% of the variance in cognitive engagement, according to the results of structural equation modeling (SEM). Work cognition and cognitive engagement accounted for half of the variance in psychological well-being among employees. Furthermore, cognitive engagement served as a partial mediator. The cognitive engagement in the health sector also provided an insight for the mediation between different variables such as in Joo et al. (2017). The theoretical structure of this study was confirmed by 290 survey replies, which established accountable artificial intelligence as a third order component. The 174 dyadic data findings further validated the participant’s cognitive engagement using accountable artificial intelligence technologies and potential value, which contributes to market efficiency, as a mediation mechanism. No prior research was available for mediating role of cognitive engagement between cognitive loading and knowledge hiding, which provided us with a gap to find the mediating relationship and we formulated the following hypothesis.

***H*_3._** *There is a significant mediation of cognitive engagement between cognitive loading and knowledge hiding.*

### Moderating Relationship of Supervisor Support

A supervisor is a person who oversees controlling and regulating students’ work in the first level of command. Supervisors keep track of how well pupils complete assignments (Stephens, 2014; Shahzad et al., 2019). Supervisors have an impact on promoting, disciplining, rewarding, altering, and other student-related actions (Swanzy, 2020; Yaghi and Bates, 2020). Supervisors in an organizational setting issue orders to employees and oversee the operations, productivity, and overall performance of a group of workers. The supervisory function, which is similar to that of a manager, has a considerable impact on building good and safe attitudes, work training, up-to-date working techniques and tactics, and spotting unfavorable workplace behavior. Supervisors generate resources such as exchanging knowledge, emotional empathy, collaboration, and helping through providing a supportive connection. Supervisors play a critical role in ensuring that students and employees are safe in the workplace (Abubakr, 2018; Al-Jaro et al., 2021).

A lot of studies have been conducted on moderating or regulating the role of supervisor support in various contexts. Achour et al. (2017) conducted research and suggested that employee well-being was inversely correlated with work family responsibilities. Management and supervisory support also increased the moderating link between work family needs and employee well-being. As a result, management and supervisory assistance played a significant moderating role in balancing job obligations and family responsibilities, as well as in improving the well-being of working female academics. Another study conducted by Achour et al. (2017) found a moderating role of supervisor support, job support, and flexibility in scheduling the work. Perceived supervisor support was found moderating between satisfaction for job, task performance, and psychological contracts (Achour et al., 2017). Similar results in a different perspective were also obtained by Wickramasinghe (2012). This allowed us to evaluate the moderating effects of supervisor support in our context of relationship between cognitive loading and the knowledge hiding. Therefore, we proposed the following.

***H*_4._** *Supervisor support effectively moderates the relationship of cognitive loading and knowledge hiding.*

**FIGURE 1:**
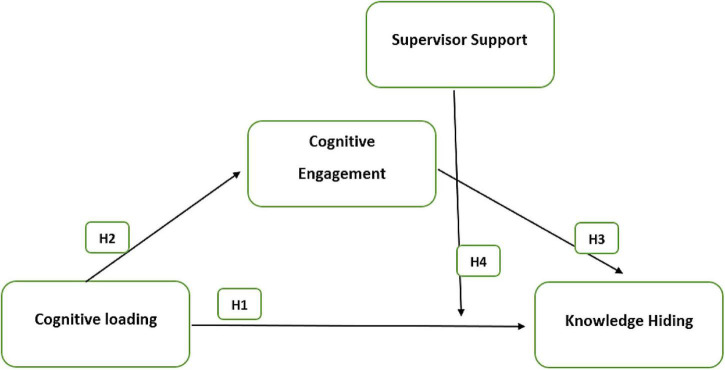
Conceptual model.

This research is based on the conceptual model (see Figure 1) for the analysis of impact of cognitive loading on knowledge hiding in virtual educational training setup with cognitive engagement in mediation and supervisor support in moderation.

## Methodology

### Participants and Procedure

This study followed a cross sectional research design and respondents were recruited under the umbrella of non-probability sampling technique through snowball sampling. Keeping in view the theoretical orientation of this study, those participants were approached who have participated in virtual trainings. Keeping in view the established criteria for suitable sample size (Xiaolong et al., 2021; Yingfei et al., 2021), a sample of 500 respondents were approached through electronically and personally administrated questionnaires. Moreover, other sample selection criteria were also considered, i.e., according to the general rule of thumb (5–10 respondents for a scale item) (Avotra et al., 2021). In this regard a total of 22 items and according to this criterion a total of 220 questionnaires were sufficient. Thus, being on the safer side we recruited respondents above the required limit. Out of these distributed questionnaires, 426 were received back and after discarding the partially filled and incomplete responses the completely filled questionnaire remained at 406 in numbers and 81% response rate. As the data were planned to be collected under cross-sectional research design through self-reported measures as there was a chance of common method bias, and thus in order to reduce the common method bias in the data, we used various techniques to reduce the issue of common method bias (Hao et al., 2020; An et al., 2021).

### Measures

This study followed a five-point Likert Scale which ranged from 5 to 1 indicating a level from strongly agree to strongly disagree. The independent variable of this study, i.e., cognitive loading, was assessed on the basis of four items borrowed from a recently developed scale of cognitive load (MCLSVE) developed by Andersen and Makransky (2021). This scale has four dimensions and from these four dimensions this study only conceptualized the one dimension related to the environment of cognitive loading. This dimension was most suited for this study and was aligned with the theoretical orientation of the study and considering the virtual trainings scenario. A sample item for this scale includes “The elements in the virtual environment made the learning very unclear”. Mediating variable of this study “cognitive engagement” was measured on the basis of a five-item scale borrowed from Cognitive Engagement Short (CE-S) developed by Greene and Miller (1996). The statements of items in this scale were reworded keeping in view the context of the study under large-scale assessment context of the participants and respondents were requested to record responses on a five-point Likert Scale. This scale measured the two dimensions of cognitive engagement (meaningful cognitive engagement and shallow cognitive engagement). Previous studies have reported a high Cronbach’s alpha value for this scale which is consistent with the previously reported standards (Greene and Miller, 1996). Similarly, the dependent variable of this study is measured based on playing dumb knowledge hiding behavior. This is the one dimension of overall knowledge hiding behavior. The other two dimensions of knowledge hiding behavior were not aligned with the theoretical orientation of this study; therefore, only one dimension of the knowledge hiding behavior was considered in this study. For this purpose we have used the well-established scale developed by Connelly et al. (2012) and recently used by Wu et al. (2022). In case of supervisor support, we have used six items to ascertain the moderation.

### Demographic Profile

Respondents were requested to report their demographic characteristics such as age, gender, and qualifications along with the job status. Demographic characteristics of the respondents indicate that most of the respondents in this regard were men and they share a major portion (i.e., 62%) while women respondents in this regard were 38%. Similarly, respondents were requested to report their educational level and data reveals that only 8.6% respondents have graduation degrees while 42.6% have master’s degree, and the remaining have other degrees (MS/MPhil and other technical degrees). While in the case of job status level, most of the respondents were on a contractual nature posting (74%), while the remaining 26% were on a permanent basis. The last demographic characteristic of the respondents pertains to their age group and results revealed that most of the respondents fall in the category of age group of 26–30 years with a higher percentage, i.e., 40%. While respondents in the age group between 30 and 35 years share a portion of 28.8%. Similarly, respondents in the age group between 20 and 25 years were 13.8% and last category having age above 35 years was 17.2%.

### Statistical Analysis

In order to test the complex nature of relationships in this study, we used the variance based structural modeling approach (partial least square) (Sarstedt et al., 2014). Thus, Smart-PLS was the best available choice for this purpose, and we have used it to test the hypothesis assumed in this research. Smart-PLS, which is based on the partial least square (PLS) approach, is the best tool that can be used where theory is less developed and in this case nothing was available regarding the relationship of cognitive loading and knowledge hiding, so it was the best fitted approach in this case (Hair et al., 2017). Moreover, PLS-SEM can easily handle the non-parametric data and thus owing to the concerns of normality this study tested a conceptualized model of this study through Smart-PLS (Hair et al., 2017).

## Data Analysis and Results

Results of SEM are assessed in two dimensions. The first dimension is related to assessment of the measurement model while the second dimension is related to measurement of the structural model. First, the measurement model is assessed for quality criteria based on reliability and validity (Hair et al., 2017). To assess reliability various measures are available, and this study used three measures to assess the reliability based on “Cronbach’s alpha, rho-A, and composite reliability. All these indicators have been found under the prescribed limit (see Table 1). In case of reliability, statistics measured through alpha for cognitive loading is 0.590. This value can be accepted as the scale for cognitive loading that is newly developed and still needs to be validated and tested, so such low values in this regard can be accepted. While for another two constructs, the value of the Cronbach alpha is above the threshold value (i.e., >0.60). Similarly, in the case of rho-A, the value for the newly developed and tested scale for cognitive loading is 0.601. Again, this value can be tested owing to the reason that this scale is tested in new contextual settings and the scale is moreover under testing.

**TABLE 1:** Reliability and validity.

Constructs	Alpha	rho-A	Composite reliability	AVE
Cognitive engagement	0.784	0.805	0.851	0.537
Cognitive loading	0.590	0.610	0.784	0.549
Knowledge hiding	0.822	0.837	0.882	0.652

While other parameters of reliability statistics, i.e., composite reliability, indicated a well-established measurement of reliability, all the values were within the acceptable range. The second portion of the assessment of the measurement model is related to checking the convergent and discriminant validity. For the measurement of convergent validity, this study has used two indicators, the first indicator of convergent validity is based on outer loadings and the other indicator of convergent validity is average variance extracted (AVE). Both criteria of convergent validity provided sufficient evidence in this regard and indicated good convergent validity (see Tables 1, 2). Initially the outer loadings values were checked, and poor outer loadings were located. Although a lower or week outer loading value was identified in cognitive engagement, however, it was retained despite being lower outer loading because the AVE of cognitive engagement was within the acceptable range (>0.50), so item CE1 was retained, despite having lower outer loading. In the case of cognitive loading item CL-3 has poor loading and it was dropped from further analysis. Finally, for the knowledge hiding outer loading value indicated good values and no item from this scale was dropped as all the indicators from this scale have good outer loadings (Mela and Kopalle, 2002).

**TABLE 2:** Outer loadings and VIF.

Constructs	Indicator	Indicator reliability	VIF
Cognitive engagement	CE1	0.586	1.365
	CE2	0.762	1.672
	CE3	0.731	1.510
	CE4	0.804	1.769
	CE5	0.760	1.492
Cognitive loading	CL1	0.674	1.110
	CL2	0.824	1.294
	CL4	0.716	1.266
Knowledge hiding	KH1	0.749	1.594
	KH2	0.873	2.331
	KH3	0.848	2.033
	KH4	0.753	1.551

Similarly, in order to assess the convergent validity, we also tested AVE and it was observed that all the constructs have AVE greather than 0.50, indicating more than 50% variance and ensuring that convergent validity has been established (see Table 1). Thus, AVE of all study constructs is above the threshold value of 0.50. For instance, in the case of cognitive engagement AVE is 0.537, for cognitive loading AVE is 0.549, and for knowledge hiding AVE is 0.652 indicating well-established convergent validity. While other indicators of discriminant validity are assessed in this study based on Fornell-Larker criterion and heterotrait-monotrait ratio of correlations (HTMT) (Hair et al., 2017; e.g., see Tables 3A,B). Initially for evaluating discriminant validity, Fornell-Larcker criterion was checked (Table 3A) and it was observed that the square root of AVE of the respective construct is greater than in the respective row and column. For instance, the square root of AVE of cognitive engagement is 0.732, which is higher than the respective values in row and column. A similar pattern is observed in the case of cognitive loading and for knowledge hiding the square root of AVE is also higher. Therefore, the first criteria of discriminant validity was established very well (Hair et al., 2011).

**TABLE 3A:** Discriminant validity (Fornell-Larker Criteria).

Construct	Cognitive engagement	Cognitive loading	Knowledge hiding
Cognitive engagement	***0.732***		
Cognitive loading	0.246	***0.741***	
Knowledge hiding	−0.374	−0.288	***0.808***

*Values in the diagonal (bold and underlined) are square root of the AVE indicating discriminant validity under Fornell-Larker Criteria.*

**TABLE 3B:** Discriminant validity (HTMT).

Construct	Cognitive engagement	Cognitive loading	Knowledge hiding
Cognitive engagement	**-**		
Cognitive loading	0.355	**-**	
Knowledge hiding	0.455	0.393	**-**

### Assessment of Structural Model

We have used a 5000 bootstrapped procedure to test the structural model to eliminate risk of non-parametric data because this procedure applies 5,000 randomly drawn sub-samples which are replaced at 0.05% (Hair et al., 2017). For the assessment of a structural model, we tested a model for *R*^2^ which is also termed as predictive accuracy. The value of *R*^2^ indicates the combined effect of variables on dependent and mediating variables. In this study, the value of the coefficient of determination has been observed to be 6% in the case of cognitive engagement and 18% in the case of knowledge hiding. It indicates that 18% change in knowledge hiding is being explained by cognitive engagement and cognitive loading. While in the case of cognitive engagement, 6% change in the mediating variable was being explained through cognitive loading (independent variable). Predictive relevance *Q*^2^ was also assessed (Hair et al., 2013) and it was observed that the value of *Q*-square was greater than the zero indicating a predictive relevance of the model (Geisser, 1975; see Table 3). Multicollinearity was also assessed to test the proper parameter estimation (Mela and Kopalle, 2002). In this study (see Table 1) all the values of variance inflation factor (VIF) were below the cutoff point, i.e., +5 and indicated that model is free from multi-collinearity (Hair et al., 2013).

**FIGURE 2:**
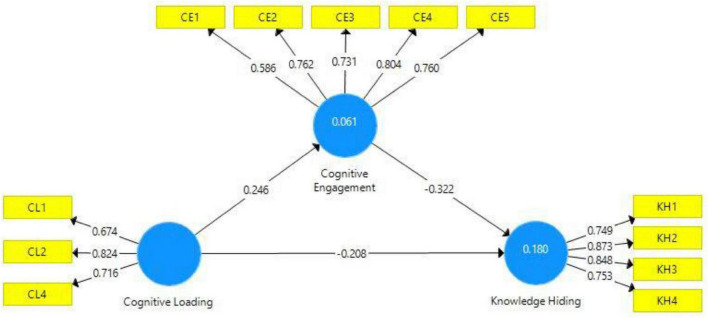
Path estimates.

The final stage to assess the structural model is based on hypothesis testing. In this regard, Table 4 presents the testing of a hypothesis. The first hypothesis of this study is related to the relationship of cognitive loading and knowledge hiding behavior. The coefficient sign (negative) indicates that cognitive loading brings a negative change in knowledge hiding. The estimated value for this path indicates that one unit change in cognitive loading will bring -0.210-unit change in knowledge hiding (Figure 2). This path is supported as *p* and *t*-statistics indicate an accepted level (see Table 4). Hence, H_1_ is supported, and it can be concluded that cognitive loadings predict knowledge hiding negatively and significantly. Similarly, H_2_ of this study is also supported/accepted as evidenced by statistical indicators. In this regard, the impact of cognitive loading on cognitive engagement has been found to be positive and significant which indicates that with the high cognitive loading there will be high cognitive engagement. The coefficient for this path indicates that one unit change in cognitive loading will bring 0.255-unit change in cognitive engagement as supported by the statistical parameters of *p* and *t* for this path and thus it can be concluded that cognitive loading has positive and significant effects on cognitive engagement. Similarly, mediation (H_3_) has been tested through the variance accounted for (VAF) approach as recommended by Hair et al. (2017). In this regard, it has been observed that direct, indirect, and total paths are significant. To calculate VAF, the indirect path is divided by total path coefficient and the outcome of this value indicates regarding mediation. If the value is less than 20%, it indicates no mediation and if the value is in the range of 20–80%, it indicates a partial mediation, while values above 80% and less than 100% depict a picture of full mediation. In our case after dividing the value of indirect effect with total effect the value yield indicates VAF of 28% which points toward a partial mediation (Cognitive Loading → Cognitive Engagement → Knowledge Hiding) and thus H_3_ is accepted here that cognitive engagement mediates the relationship between the cognitive loading and knowledge hiding behaviors of the trainees (see Table 5).

**TABLE 4:** Hypothesis testing.

Direct hypothesis		β	*t*	*p*	Status
**H1**	Cognitive loading → Knowledge hiding	−0.210	4.170	0.00	Supported
**H2**	Cognitive loading → Cognitive engagement	0.255	4.683	0.00	Supported

**Mediation hypotheses**		**Indirect effect**	**Total effect**	**VAF**	**Status**

**H3**	Cognitive loading → Cognitive engagement → Knowledge hiding	−0.083	−0.293	28%	Supported

**TABLE 5:** Direct and indirect paths.

Direct paths			

Path	Coefficient	*t*	*p*
Cognitive engagement → Knowledge hiding	–0.326	6.890	0.000
Cognitive loading → Cognitive engagement	0.255	4.683	0.000
Cognitive loading → Knowledge hiding	–.210	4.170	0.000
**Indirect path**			
Cognitive loading → Cognitive engagement → Knowledge hiding	–0.083	3.824	0.000
**Total path**			
Cognitive loading → Cognitive engagement → Knowledge hiding	–0.293	6.001	0.000

Moderation analysis has been done through the process macro (see Table 6). It was conceptualized that if the perceived supervisor support is high and cognitive engagement is also high it will lead toward a mechanism to reduce the knowledge hiding more strongly. For this purpose, it was hypothesized that cognitive loading will increase the cognitive engagement, and if at this stage perceived supervisor support is also high it will reduce the knowledge hiding more rigorously.

**TABLE 6:** Moderation analysis.

Model summary						
***R***	***R*-sq**	**MSE**	***F***	**df1**	**df2**	***p***

0.2406	0.0579	0.6819	24.8277	1.0000	404.0000	0.0000

Model						

	**Coeff**	**se**	**t**	***p***	**LLCI**	**ULCI**

Constant	1.9025	0.1298	14.6554	0.0000	1.6473	2.1577
CL	0.2212	0.0444	4.9827	0.0000	0.1339	0.3084

Model summary						

***R***	***R*-sq**	**MSE**	***F***	**df1**	**df2**	***p***

0.4493	0.2018	0.6350	25.3498	4.0000	401.0000	0.0000

Model						

	**Coeff**	**se**	**t**	***p***	**LLCI**	**ULCI**

Constant	3.8589	0.6151	6.2734	0.0000	2.6496	5.0681
CL	−0.1867	0.0442	−4.2203	0.0000	−2737	−0.0997
CE	−0.3725	0.2090	−1.7821	0.0755	−0.7834	0.0384
PSS	0.1651	0.1546	1.0679	0.2862	−0.1388	0.4691
Int_1	0.0376	0.0546	0.6892	0.4911	−0.0697	0.1450

Test(s) of highest order unconditional interaction(s)						

	**R2-chng**	***F***	**df1**	**df2**		***p***

M*W	.0009	.4750	1.0000	401.0000		.4911

Index of moderated mediation						

	**Index**	**BootSE**	**BootLLCI**		**BootULCI**	

PSS	.0083	.0130	−.0183		.0335	

## Discussion

This research is based on certain factors which result into knowledge hiding in different ways. The factor of cognitive loading was derived from the cognitive load theory by Sweller (1988). Another factor which could mediate the relationship of cognitive load and knowledge hiding was derived from ICAP theory of cognitive engagement by Chi (2009). Moderating the role of supervisor support was also analyzed in this study regulating the relationship of cognitive loading and knowledge hiding. The research was conducted to identify the impact of cognitive loading on knowledge hiding behaviors in the context of virtual educational trainings. The outcomes of this research revealed some interesting results. Our first hypothesis, which was about the relationship of cognitive loading on knowledge hiding, was accepted. The reason behind such a relationship is the loading of the cognitive segment of the brain with overflowing knowledge in virtual education. The medium used in such kind of trainings is either online or through digital sources. In such mediums, a trainer is unable to deliver all the information to the knowledge seekers.

This thing leads to hiding of some of the knowledge especially in cases of rational knowledge hiding in which a knowledge source hides the information purposely or has justifications for the knowledge hiding. This concept of knowledge hiding was first given by Connelly et al. (2012). So, it was obvious to get such kind of results. These kind of results are in accordance with many researchers of the past where occupancy of the brain leads to knowledge hiding as in our first hypothesis (Babic et al., 2019). Our second hypothesis was about the relationship of cognitive load and cognitive engagement. This hypothesis was also accepted as cognitive engagement is derived from the ICAP theory of cognitive engagement which deals with the interactive ability of learners (Chi and Wylie, 2014). This factor is directing the brain possession with the knowledge or the information provided by the trainer, so working memory is full or loaded at a time when getting trainings either physically or online. Similar kinds of results were obtained in previous studies in different contexts regarding students’ cognitive engagement (Carini et al., 2006).

Our third hypothesis was about mediating the role of cognitive engagement between cognitive loading and knowledge hiding. This hypothesis was also supported in this setup of research showing the supporting impact of cognitive engagement between the two factors. Such kinds of results are obtained due to the reason that cognitive loading could be helped through cognitive engagement in knowledge hiding in the virtual educational training. If the working memory is loaded with all the information to be delivered and is engaged in delivering all information to the knowledge seekers, then there would be certain knowledge hiding in the setup. Mediating the role of cognitive engagement has also been previously studied by many researchers who obtained similar results of positive mediation of cognitive engagement in different perspectives (Cheng, 2013; Joo et al., 2017; Raja Kamal et al., 2021).

The fourth and the last hypothesis was about moderating the role of supervisor support between cognitive loading and knowledge hiding. This was also accepted as supervisors and their support is necessary in obtaining desired results (Shahzad et al., 2019). Supervisor support is necessary in learning in every medium and is reported by many (Jensen and Solheim, 2020; Swanzy, 2020; Yaghi and Bates, 2020; Gasa and Gumbo, 2021). The supervisor support could regulate the relationship of cognitive loading and the knowledge hiding as supervisors could guide what to share and what not to from the available knowledge load in the working memory of the learners. The results are in line with many previous researchers who analyzed the moderating roles of supervisor support. The moderating role of supervisor support has been reported by Achour et al. (2017). Overall results of this research provide a significant contribution in understanding the contributing factors of knowledge hiding.

## Theoretical Contributions

The study has certain theoretical contributions. First, no prior study has attempted to check the impact of cognitive loadings and cognitive engagements from trainers. Second, the knowledge hiding behavior of the trainers has been found to be an important factor in the role of supervisor support between cognitive loading and knowledge hiding. Third, the aim of this study is to document a new predictor of knowledge hiding from the perspective of trainers and trainees in virtual trainings and this study tends to add new theoretical insights.

## Practical Implications

This study has many implications in the real ecosphere. For instance, this study will be helpful, and it can be recommended and suggested that in virtual trainings, practitioners should increase the interactions of the trainees with trainers and their colleagues so that it should become a source to decrease the knowledge hiding in organizational courses and its circuits. Second, it has been empirically initiated that cognitive loadings lead toward cognitive engagement which is also an important outcome and stresses upon the fact that if the trainers create an interaction environment for the trainees it brings cognitive engagement among the trainees and they become engaged cognitively. So cognitive loading has double benefits for the trainers and trainees without instructor involvement in rationalized knowledge hiding. Organizations can ensure the maximum sharing of knowledge by being involved in positive reinforcement behavior to motivate the instructors.

## Limitations and Future Research

The present study has certain limitations. (1) It has a sample size of 500 which is a small sample concerning the population of the students. Future studies should be conducted with a large sample size and probability sampling to ensure rigor in the study. This study can be replicated with more mediating variables (like understanding behavior, cognitive interest, etc.) and moderating variables (gender, technical support, supervisor motivation, etc.). This study is conducted in Chinese universities; it should be conducted in other countries of the world to ensure the generalization of the results.

## Conclusion

Based on empirical findings of this it can be safely concluded that cognitive loading has the potency to affect the knowledge hiding behavior of Art design employees. Keeping in view the theoretical orientation of this study as data were collected from the respondents who have participated in Art design virtual training’s as it has been confirmed that if the interaction in virtual trainings is high it increases the cognitive loadings of the trainers in virtual trainings and boosts them and motivates them to avoid knowledge hiding. Thus, it can be recommended that in Art design virtual trainings practitioners should increase the interactions of the trainees with trainers and their colleagues so that it should become a source to decrease the knowledge hiding in organizational circuits. Second, it has been empirically found that cognitive loadings lead toward cognitive engagement which is also an important outcome and stresses upon the fact that if the trainers create an interaction environment for the trainees it brings cognitive engagement among the trainees, and they become engaged cognitively. So cognitive loading has double benefits for the trainers and trainees. Moreover, it has been found that cognitive engagement plays a mediating role between the relationships of cognitive loading and knowledge hiding. Simply, cognitive engagement carries out the effect of cognitive loading and it passes this to the knowledge hiding behavior of the trainees.

## Data Availability Statement

The original contributions presented in the study are included in the article/supplementary material, further inquiries can be directed to the corresponding author.

## Ethics Statement

The studies involving human participants were reviewed and approved by Jiangxi Normal University of Science and Technology. The patients/participants provided their written informed consent to participate in this study. The study was conducted in accordance with the Declaration of Helsinki.

## Author Contributions

TG: drafting the manuscript and data collection. LK: reviewing, analysis, and supervision. Both authors contributed to the article and approved the submitted version.

## Conflict of Interest

The authors declare that the research was conducted in the absence of any commercial or financial relationships that could be construed as a potential conflict of interest.

## Publisher’s Note

All claims expressed in this article are solely those of the authors and do not necessarily represent those of their affiliated organizations, or those of the publisher, the editors and the reviewers. Any product that may be evaluated in this article, or claim that may be made by its manufacturer, is not guaranteed or endorsed by the publisher.
